# Outlining the Roles of Membrane-Foulant and Foulant-Foulant Interactions in Organic Fouling During Microfiltration and Ultrafiltration: A Mini-Review

**DOI:** 10.3389/fchem.2020.00417

**Published:** 2020-06-03

**Authors:** Hao Xu, Kang Xiao, Xiaomao Wang, Shuai Liang, Chunhai Wei, Xianghua Wen, Xia Huang

**Affiliations:** ^1^School of Civil Engineering, Guangzhou University, Guangzhou, China; ^2^College of Resources and Environment, University of Chinese Academy of Sciences, Beijing, China; ^3^Center for Ocean Mega-Science, Chinese Academy of Sciences, Qingdao, China; ^4^State Key Joint Laboratory of Environment Simulation and Pollution Control, School of Environment, Tsinghua University, Beijing, China; ^5^College of Environmental Science and Engineering, Beijing Forestry University, Beijing, China; ^6^Research and Application Center for Membrane Technology, School of Environment, Tsinghua University, Beijing, China

**Keywords:** membrane fouling, intermolecular interaction, non-covalent interaction, covalent interaction, steric effect

## Abstract

Membrane fouling remains a notorious problem in microfiltration (MF) and ultrafiltration (UF), and a systematic understanding of the fouling mechanisms is fundamental for solving this problem. Given a wide assortment of fouling studies in the literature, it is essential that the numerous pieces of information on this topic could be clearly compiled. In this review, we outline the roles of membrane-foulant and foulant-foulant intermolecular interactions in MF/UF organic fouling. The membrane-foulant interactions govern the initial pore blocking and adsorption stage, whereas the foulant-foulant interactions prevail in the subsequent build-up of a surface foulant layer (e.g., a gel layer). We classify the interactions into non-covalent interactions (e.g., hydrophobic and electrostatic interactions), covalent interactions (e.g., metal-organic complexation), and spatial effects (related to pore structure, surface morphology, and foulants size for instance). They have either short- or long-range influences on the transportation and immobilization of the foulant toward the membrane. Specifically, we profile the individual impacts and interplay between the different interactions along the fouling stages. Finally, anti-fouling strategies are discussed for a targeted control of the membrane-foulant and foulant-foulant interactions.

## Introduction

Microfiltration (MF) and ultrafiltration (UF) membrane separation technologies are playing an increasingly important role in water purification, wastewater treatment, and resource recovery (Baker, 2012; Tong et al., 2019; Xiao et al., 2019b). However, membrane fouling remains a notorious problem in MF/UF processes (Guo et al., 2012; Shi et al., 2014; Meng et al., 2017). Membrane fouling causes higher filtration resistance and lower separation efficiency. To mitigate fouling, intensive hydraulic regulation (such as air scouring and crossflow circulation) and frequent chemical cleaning not only consume a large amount of energy and cleaning agents, but also shorten the membrane life and increase the depreciation cost (Porcelli and Judd, 2010; Wei et al., 2011; Shi et al., 2014; Xiao et al., 2019b). Organics are a major group of foulant, and the fouling caused by organics, called organic fouling, merits particular attention in MF/UF operation (Lee et al., 2007; Guo et al., 2012). For a more cost-effective and targeted control of membrane fouling, it is necessary to clearly understand the reasons, factors, and dynamics of fouling. Membrane-foulant and foulant-foulant interactions are the fundamental principles of membrane fouling (Wang and Waite, 2008; Lin et al., 2010; Tang et al., 2011; Xiao et al., 2011). These interactions can be classified into non-covalent interactions, covalent interactions, and spatial effects, which may correspond to different physical/chemical means to combat them. This review aims to concisely outline the prevailing mechanisms and the targeted control strategies of membrane fouling from the perspective of membrane-foulant and foulant-foulant interactions.

## Membrane-Foulant Interaction

**Figure 1:**
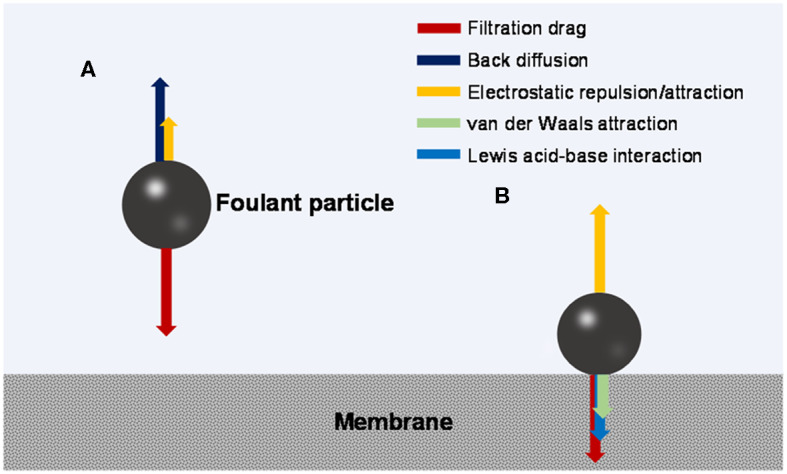
Force analysis of a foulant particle **(A)** moving toward the membrane and **(B)** sitting on the membrane surface.

During the fouling process, an organic foulant particle from the bulk solution travels through a possibly existing concentration polarization (CP) boundary layer and arrives at the membrane surface (outer surface or pore walls). The mass transfer may be influenced by hydrodynamic effects such as advection driven by filtrational flow, Brownian diffusion, shearing-induced diffusion, and/or inertial lift, depending on the size of the foulant particle (Belfort et al., 1994). In the CP layer, the mass transfer is hindered by the chemical potential gradient that is also a function of foulant-foulant interaction (Wang et al., 2011; Wang and Li, 2012). The foulant particle may also receive electrostatic long-range attractive or repulsive force from the membrane. Figure 1A illustrates the possible force balance in the CP layer.

At the membrane surface, non-covalent and covalent interactions may occur between the membrane and foulant, and may be influenced by spatial effects (Zhang and Song, 2000; Maximous et al., 2009; Huang et al., 2014; Wang et al., 2015). Among them, non-covalent interactions mainly include hydrophobic interaction and electrostatic interaction (van Oss, 2006), both of which are different forms of electromagnetic interaction with varied strength and effective distance (long- or short-range). Figure 1B illustrates the possible force balance at the membrane-foulant interface.

### Non-covalent Interaction

Non-covalent interactions include van der Waals interactions, Lewis acid-base interactions, electrostatic interactions, and random thermal motions. In the framework of the extended DLVO (XDLVO) theory, hydrogen bonding is regarded as a generalized Lewis acid-base interaction; as such, van der Waals and Lewis acid-base interactions together are classified as hydrophobic interaction (van Oss, 2006). In MF/UF water treatment systems, the hydrophobicity and surface charge (potential) of the membrane and foulant are two important factors for the membrane-foulant non-covalent interaction, corresponding to hydrophobic adsorption and electrostatic attraction/repulsion, respectively (Weis et al., 2005; van Oss, 2006; Maximous et al., 2009; Meng et al., 2009; Xiao et al., 2014b).

#### Electrostatic Interaction

The membrane-foulant electrostatic interaction could be either attractive or repulsive, depending on the sign of the charges carried by the membrane and foulant. Opposite charges promote transport and adsorption of the foulants to the membrane, whereas the same charges do not (Zhan et al., 2004; Weis et al., 2005; Cai et al., 2016). At different pH conditions, the sign and amount of charge may be different due to protonation/deprotonation of the functional groups. For a protein moving toward the membrane with the same sign of charge, the farther the pH is from the isoelectric point, the higher surface charge density the protein carries, and the stronger electrostatic repulsion it will receive from the membrane (Cai et al., 2016); as a result, this allows for a higher quasi-steady state flux (corresponding to a stronger filtration drag against the electrostatic repulsion) at force balance (Palecek and Zydney, 1994). The amount of surface charges carried by the membrane or foulant may also vary with ionic strength or hardness ion concentration due to adsorption of the ions (Wang Z. et al., 2018; You et al., 2020). Zeta potential is usually used to characterize the apparent potential on the water film-covered surface (the water film is tightly bound to the surface when moving in aqueous media) (Kim et al., 1997; Wang et al., 2000; Hunter, 2013). Aspects of the solution environment, such as ionic strength, can affect the gradient of electric potential across the water film and thus affect the zeta potential (Hunter, 2013; Israelachvili, 2015). Approximately, the membrane-foulant electrostatic interaction energy could be related to the product of the zeta potentials of the membrane and foulant, which reflects the combined effect of the two (Xiao et al., 2011; Cai et al., 2016).

#### Hydrophobic Interaction

The membrane-foulant hydrophobic interaction includes van der Waals and Lewis acid-base interactions. Hydrogen bonding could be classified as a generalized Lewis acid-base interaction; as such, the hydrophobic interaction is usually dominated by the Lewis acid-base interaction (van Oss, 2006). The hydrophobic behavior could be understood from the perspective of hydrogen bonding. Immersion of a hydrophobic surface (such as that of a membrane or a foulant particle with a low density of hydrogen bonding sites) in water will disturb the original dense network of hydrogen bonds of water, reducing the number of hydrogen bonds, or distorting the hydrogen bonds, and thus increase the free energy at the enthalpic or entropic level (Chandler, 2005). Therefore, the surrounding water molecules will spontaneously push the hydrophobic surface together to reduce the water-contacting interface area, and this phenomenon is apparently observed as hydrophobic attraction. In the XDLVO theory, the hydrophobic effect due to hydrogen bonding is incorporated into the Lewis acid-base term as an extension of the DLVO theory (van Oss, 2006). The membrane-foulant hydrophobic adsorption mainly affects initial fouling or irreversible fouling, for which the foulant particle directly contacts the membrane surface.

A large number of studies have shown that a membrane surface with higher hydrophobicity would suffer more serious adsorptive fouling (Jin et al., 2001; Weis et al., 2005; Maximous et al., 2009; Xiao et al., 2014b; Zhao et al., 2018). Higher hydrophobicity of the foulant would make the hydrophobic adsorption stronger (Xiao et al., 2014b; Mu et al., 2019). The air-water-solid three-phase contact angle (θ) is usually used to judge the relative hydrophobicity of the membrane surface or foulant particle surface, and a smaller contact angle suggests a weaker hydrophobicity (or stronger hydrophilicity) (van Oss, 2006; Israelachvili, 2015; Jiang and Patel, 2019). The interference of surface roughness and porosity in contact angle measurement could be corrected using, e.g., the Cassie-Baxter relation (Han et al., 2019a,b). Approximately, the membrane-foulant hydrophobic interaction energy could be related to the sum of the cosθ's of the membrane and foulant, which reflects the combined effect of the membrane and foulant hydrophobicity (Xiao et al., 2011).

#### Identification of the Dominant Mechanism

In MF/UF systems, there has long been a controversy over whether the hydrophobic or the electrostatic effect dominates the membrane-foulant non-covalent interaction. Researchers have studied not only the individual impact of membrane/foulant hydrophobicity/surface charge on membrane fouling, but also the joint impact of them in different combinations, such as the combined effects of membrane and foulant surface charge (Xiao et al., 2014b), foulant hydrophobicity/surface charge and membrane hydrophobicity (Yu et al., 2018), and foulant hydrophobicity and membrane surface charge (Raspati et al., 2011). Xiao et al. (2011) derived a semi-empirical multiple regression model based on the XDLVO theory, describing the combined effect of the hydrophobic and electrostatic properties (represented by water contact angle and zeta potential, respectively) of the membrane and foulant on the adsorption equilibrium constant. Statistical analysis showed that the contact angle term (sum of the cosθ's) was significant, whereas the zeta potential term (product of the membrane and foulant zeta potentials) was not, indicating that the main mechanism for adsorptive fouling of MF/UF membranes might be hydrophobic interaction rather than electrostatic interaction (Xiao et al., 2011). However, for further accurate quantification, the impacts of surface roughness (Hoek and Agarwal, 2006), molecular conformational changes (Nakanishi et al., 2001), or entropic repulsion (Grasso et al., 2002) should be rigorously considered. Future studies on the leading mechanisms could adopt mechanistic models with comprehensive consideration of the factors, statistical models covering a wider range of the variables and samples, or semi-empirical computational chemistry approaches such as molecular dynamics and molecular docking (Shaikh et al., 2018; Liu et al., 2019).

### Covalent Interaction

Covalent interactions, such as the metal-organic complexation, could also occur between the membrane and foulant. The functional groups (e.g., carboxyls), present on the foulant particle and membrane surfaces, can be bridged by complexation with multivalent metal ions such as calcium and magnesium ions (Mo et al., 2012; Wang et al., 2015; Xin et al., 2015). The covalently adsorbed foulant could serve as an initial riveting layer on the membrane surface, laying a foundation for subsequent gel layer build-up. The gel layer is a network of foulant molecular chains which are also linked by metal ion-mediated complexation (Wang and Waite, 2009; Chen et al., 2016). The type and concentration of metal ions (Wang et al., 2015; Xin et al., 2015) as well as the type and density of organic ligands (Wang and Waite, 2009; Guo et al., 2012; Xiao et al., 2014b) can affect the complexation, as evidenced by atomic force microscope (AFM) and quartz-crystal microbalance with dissipation monitoring (QCM-D) measurements (Contreras et al., 2011; Mo et al., 2012). There is a critical concentration for Ca^2+^ in the feed solution, below which the initial fouling rate (flux decline rate) increases with the increase of the Ca^2+^ concentration; above it, the initial fouling rate decreases because excessively high Ca^2+^ concentrations cause the foulant particles to be bridged together in the feed solution before arriving at the membrane surface, so that the foulants form loose flocs instead of a dense adsorption layer on the membrane surface (Mo et al., 2011).

### Spatial Effects

The membrane pore structure (e.g., porosity and pore shape) and surface morphology (e.g., roughness) and the foulant size and morphology can have spatial effects on membrane fouling (Le-Clech et al., 2006; Fu et al., 2008; Xiao et al., 2014a; Kumar and Ismail, 2015; Cai et al., 2018; Li et al., 2019). The complexity of the pore structure affects fouling in many ways: on the one hand, the filtration flux of a membrane with straight-through pores decreases sharply due to pore blockage, whereas the flux of a membrane with highly interconnected pores decreases mildly due to that the fluid can easily bypass the blocked point (Ho and Zydney, 1999, 2006); on the other hand, highly crosslinked porous network structures are easier to catch and intercept foulant particles (especially the foulant particles with irregular shapes) and are more likely to suffer internal fouling (Xiao et al., 2014a; Fan et al., 2018). The membrane surface roughness can affect fouling at different scales. At the micrometer scale, the topography of membrane surface affects the microflow, such that the foulant particles are more prone to deposit in the “valleys” than on the “hills” (Kang et al., 2006; Hashino et al., 2011; Won et al., 2016). At the nanometer scale, the surface roughness can affect the interfacial interaction between the membrane and foulant (Hoek et al., 2003; Zhao et al., 2015). Empirically, a smoother membrane surface corresponds to a slower initial fouling, but probably a higher irreversibility against hydraulic washing once fouling occurs (Oh et al., 2009; Jin et al., 2010; Wang and Tang, 2011).

The spatial effects on the non-covalent interaction are represented by the effect of membrane pore structure and surface roughness on foulant adsorption (Xiao et al., 2014a; Zhao et al., 2015; Fan et al., 2018). Compared with membranes with perforated plate-like (e.g., PCTE) or particulate bed-like morphologies (e.g., PVDF), fibrous mesh-like membranes (e.g., PTFE) are beneficial for reducing hydrophobic adsorption (Xiao et al., 2014a; Fan et al., 2018). This is because thin, fiber-like pore walls provide limited contactable area for the adsorption, and the foulant particles sitting on the fibers are not stable under hydraulic disturbance. Moreover, interfacial force calculation suggests that the hydrophobic attractive force of a foulant particle received from a thread-like (or cylindrical) membrane object is weaker than that from a plane-like membrane object at the same distance (Fan et al., 2018). At the micrometer scale, the membrane surface roughness can influence the force balance of a foulant particle sitting on the membrane surface. Higher roughness is conducive to the leverage effect for the stripping or rolling of the particle under hydrodynamic shear (Hong et al., 2014). At the nanometer scale, the roughness can obstruct close contact between the membrane and foulant surfaces, increasing the effective distance and weakening the force between them (especially for short-range forces which decay steeply with increasing distance) (Hoek et al., 2003; Hoek and Agarwal, 2006).

On the other hand, non-covalent and covalent interactions can alter spatial effects such as the membrane pore sieving effect during the development of membrane fouling (Xiao et al., 2014b). At the initial stage of fouling, hydrophobic adsorption of the foulant onto the pore walls can narrow the pore channels and in turn promote the mechanical interception of the foulant particles at the pore openings. A gel layer will then be formed when the foulant intercepted on the membrane surface reaches a critical concentration (i.e., gel point) (Xiao et al., 2013). Therefore, hydrophobic adsorption accelerates this process. The membrane-foulant complexation is also beneficial to the accumulation of foulant on the membrane surface, composing a premise layer to promote subsequent growth of the gel layer (Mo et al., 2012; Chen et al., 2018).

### Summary

**Figure 2:**
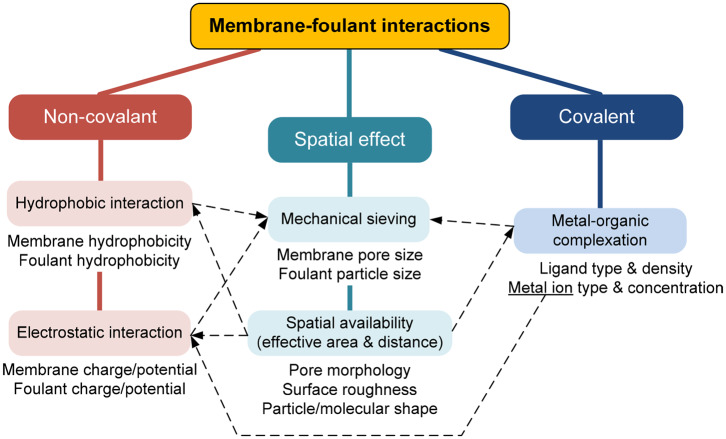
Relationships among membrane-foulant interactions.

The relationships among the membrane-foulant non-covalent, covalent, and spatial effects are schematically summarized in Figure 2. A conceptual model for a combination of these effects might be expressed in the form of:

(1) $$\begin{array}{r} E_{\text{T}}=\sum E_{i}S_{i}+\varepsilon =\;\left(E_{\text{HP}}S_{\text{HP}}+E_{\text{EL}}S_{\text{EL}}+E_{\text{CV}}S_{\text{CV}}+\ldots \right)\;+\varepsilon \end{array}$$

where *E*_T_ is the total effect (e.g., the total interaction energy); *E*_*i*_'s are the components represented by hydrophobic interaction (HP), electrostatic interaction (EL), covalent interaction (CV), etc.; *S*_*i*_'s are the correction factors for spatial effects; and ε is the error term due to other marginal effects and non-linearity of the expression. *E*_HP_ and *E*_EL_ are approximately proportional to (cosθ_m_ + cosθ_f_) and ζ_m_ζ_f_, respectively, where θ and ζ are the water contact angle and zeta potential of the membrane (m) and foulant (f) according to a derivation from the XDLVO theory (Xiao et al., 2011). *E*_CV_ may be related to the strength and density of the metal-organic bonds for the complexation. *S*_*i*_ may be a function of the effective contacting area and/or distance.

## Foulant-Foulant Interaction

When the bare membrane surface is completely covered (shielded) by the foulant material after the initial adsorption and pore blocking, further development of a foulant layer on the outer surface would be controlled by the foulant-foulant cohesion rather than the foulant-membrane adhesion (Wang et al., 2013; Huang et al., 2014; Xiao et al., 2014b).

### Covalent Interaction

Metal-organic complexation is the critical mechanism for gel layer formation. The skeleton of the gel matrix is constituted by the polymers of, e.g., polysaccharides and humic acids (Yang N. N. et al., 2011; Xiao et al., 2014b; Chen et al., 2016). The thermodynamics of gel formation can be described by the Flory-Huggins theory for polymeric systems (Lei et al., 2016). In general, the gel layer has a quite high porosity (and water content) but extremely low water permeability. For example, the calcium alginate gel layer (4 mmol Ca/g alginate) has a porosity of 0.97 and a high specific filtration resistance of 10^19^ m^−2^, and the calcium humate gel layer (5 mmol Ca/g humic acid) has a porosity of 0.99 and a specific resistance of 4 × 10^18^ m^−2^, under a constant pressure of 100 kPa (Wang and Waite, 2008; Yang N. N. et al., 2011). The reason is that the water contained in the gel layer is mainly bound water and the migration of free water is extremely limited. As water passes through the gel layer, the water molecules are dragged from the gel layer (with a low chemical potential) to the permeate side (with a much higher chemical potential), so that a high transmembrane pressure is required to balance the gap of chemical potential (Hong et al., 2014; Chen et al., 2016).

The three-dimensional network structure of the gel layer is formed by polymer chains crosslinked by multivalent metal ions via complexation (Wang and Waite, 2008; Chen et al., 2016). The specific resistance of the gel layer is closely related to the degree of crosslinking and complexation. The polymer chain structure, ligand type and density, and metal ion type and concentration could be important factors for the complexation. The equilibrium constant for Ca-humic acid complexation was reported to be ~1 × 10^3^ L/mol (as determined by isothermal titration calorimetry) (Wang et al., 2015), while for Ca-microbial products it was ~5 × 10^3^ L/mol (Wang and Waite, 2009) or ~1.5 × 10^4^ L/mol (Xiao et al., 2014b) (as determined by complexometric titration) in membrane bioreactor (MBR) systems; relatively strong carboxylic sites (with pKa <5.5 or 6) were most responsible for the complexation.

Calculation based on the density functional theory (DFT), a computational chemistry approach, indicates that intermolecular Ca-alginate complexation occurs more preferentially than intramolecular complexation, and the two alginate chains connected by Ca^2+^ tend to stretch in a tetrahedral angle (cross to each other) rather than parallel to each other, which homogenizes the gel matrix and eventually form an “egg-box” structure (Zhang et al., 2018). The intermolecular bridging of alginate chains is enhanced significantly by the increase of Ca^2+^ concentration (Bruus et al., 1992; Zhang et al., 2017). Compared with Mg^2+^ and Fe^3+^, Ca^2+^ has been well-reported to be able to form a highly porous but poorly permeable alginate gel layer (Davis et al., 2003; Wang and Waite, 2009).

### Non-covalent Interaction

As the molecular chains in the gel layer are normally negatively charged, the electrostatic repulsion would affect the inter-chain distance and, hence, the density and uniformity of the gel layer. The charge density is related to the ionization of acid groups (such as carboxyls) that is a function of pH (Kratz et al., 2000). At lower pH, the acid groups are less charged and the electrostatic repulsion weakens, resulting in deteriorated fouling (Chan and Chen, 2001). The increase in ionic strength of the solution can also reduce the electrostatic repulsion by compressing the electric double layer (Hunter, 2013; Israelachvili, 2015; You et al., 2020). When there is free Ca^2+^ in the solution, it can also compress the double layer and enable a closer contact between the chains.

When multiple foulants coexist in the solution, the non-gelling foulant may be adsorbed on the surface or inside the gel layer through non-covalent interactions such as hydrophobic and electrostatic interactions. For example, proteins could be adsorbed and penetrate deep into the Ca-alginate gel layer (Wang and Waite, 2008) and form a protein-alginate composite foulant layer (Pendashteh et al., 2011). In an organic-inorganic composite foulant layer (such as a polysaccharide gel blended with silica and metal oxides), the non-covalent adsorption (such as electrostatic attraction of opposite charges) or covalent bridging between the inorganic particles and organic polymer backbones will increase the compactness of the foulant layer (You et al., 2005; Meng et al., 2007; Chen et al., 2018).

### Spatial Effects

When the gel layer is blended with inorganic particles (e.g., SiO_2_, Al_2_O_3_, Fe_2_O_3_, and kaolin), the inorganic particles can affect the structure, permeability, and filtration resistance of the gel layer through hydraulic effect and physical/chemical adsorption (Ao et al., 2018; Chen et al., 2018; Ma B. W. et al., 2019). Blending of impermeable solid particles would lower the overall permeability of the gel layer; however, when the content of inorganic particles is high enough to interrupt the continuous gel structure (creating gaps around the particles), the water permeability would be largely elevated (Chen et al., 2018). The gaps are also encouraged by the adhesion between inorganic particles and organic polymer: the stronger adsorption, the more likely organics tend to agglomerate at the surface of the inorganic particles rather than to form a continuous gel layer (Chen et al., 2018). The adsorption could be contributed by hydrophobic interaction, electrostatic attraction, and/or covalent complexation between the gelling polymer and inorganic particles (Giese and van Oss, 2002; Israelachvili, 2015; Chen et al., 2018). According to Chen et al. (2018), a conceptual model for the permeability of the composite gel/cake layer might be expressed in the form of:

(2) $$\begin{array}{r} J_{\text{T}}=\sum J_{i}f_{i}\left(K\right)\;+\varepsilon =\;\left(J_{\text{OG}}f_{\text{OG}}+J_{\text{IP}}f_{\text{IP}}+J_{0}f_{0}\right)\;+\varepsilon \end{array}$$

where *J*_T_ is the overall permeability contributed by the components of organic gel (*J*_OG_), inorganic particles (*J*_IP_), and gaps filled with water (*J*_0_); *f*_*i*_ is a distribution function depending on the adsorption parameter *K*; and ε is the error term.

## Role of the Interactions at Different Fouling Stages

**Figure 3:**
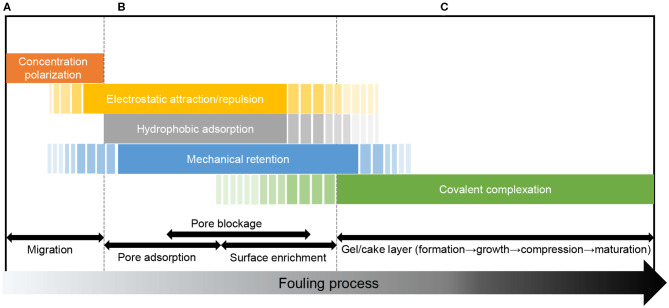
Membrane-foulant and foulant-foulant interactions at different fouling stages. **(A)** Pre-fouling stage, **(B)** Membrane adsorption/ blocking stage, **(C)** Gel/cake layer stage.

The interactions specific to different fouling stages are schematically summarized in Figure 3.

### (a) The Pre-fouling Stage

When migrating from the bulk solution toward the membrane, the foulant particle is dragged by the filtration flow but repelled by the concentration gradient in the CP boundary layer (i.e., a foulant-foulant repulsion in a broad sense) (Wang et al., 2011; Wang and Li, 2012; Xiao et al., 2013), and the CP phenomenon stems from the mechanical rejection of the foulant by the membrane (a steric effect). The foulant in the CP layer may also be attracted or repelled by long-range electrostatic force from the membrane surface (membrane-foulant non-covalent interaction). If the particle is micron-sized, it may also undergo hydrodynamic effects (e.g., shear-induced back transport or inertial lift) in the boundary layer (Belfort et al., 1994).

### (b) The Membrane Adsorption/Pore Blocking Stage

After the foulant arrives at the membrane surface or pore walls, the membrane-foulant interaction plays a governing role. Hydrophobic, electrostatic, or covalent adsorption of the foulant onto the pore walls would narrow the pore channels, which enhances the size exclusion effect and promotes mechanical rejection of subsequent foulant particles (spatial effect). The increased rejection rate would in turn promote concentration polarization above the membrane surface (influencing subsequent foulant transport) (Song and Elimelech, 1995; Wang et al., 2011). The porosity and pore morphology changed by the pore adsorption would vary the local fluid conditions (e.g., local flux) near the pores (Ho and Zydney, 1999), or have a feedback effect on the adsorption by altering the effective area or distance of the interaction (Fan et al., 2018). The non-covalent or covalent adsorption on the membrane surface, as well as the mechanical retention at the pore openings, contributes to the accumulation of foulant concentration on the membrane surface (Xiao et al., 2014b). The membrane-foulant interactions are closely related to the hydrophobicity, charge, functional groups (e.g., complexing groups), and spatial properties (e.g., pore structure, surface roughness, and particle size) of the membrane and foulant.

### (c) The Gel/Cake Layer Stage

When the foulant accumulated on the membrane surface reaches a critical concentration (e.g., the gel point), a gel layer begins to form and shield the membrane surface (Wang and Waite, 2008; Yang N. N. et al., 2011; Xiao et al., 2013). The subsequent foulant from the feed solution mainly interacts with the gel/cake layer surface rather than the membrane surface, and the main effect shifts from membrane-foulant interaction to foulant-foulant interaction. The covalent complexation is the key mechanism for gel layer constitution (Wang and Waite, 2009; Chen et al., 2016), while the non-covalent and steric effects could influence the porosity, compactness, and permeability of the foulant layer (Chen et al., 2018). These effects are closely related to the hydrophobicity, charge, size, morphology, and functional groups of the foulant components.

There are a number of mathematical models describing the process and mechanisms of membrane fouling. Process models describe the variation of flux (or resistance) as a function of filtration time (or volume). The resistance-in-series model divides the total resistance into membrane resistance, pore blocking resistance, surface foulant layer resistance, etc., based on the spatial distribution of the foulant (Yeh and Cheng, 1993). According to the temporal change of the resistance, the filtration laws classify the fouling modes into standard blocking (on the pore walls, including fast and slow adsorption modes), complete blocking (at the pore opening), intermediate blocking (random coverage of the porous surface), and cake filtration (for cake/gel layer growth) (Hlavacek and Bouchet, 1993; Bowen et al., 1995; Xiao et al., 2019a). Some researchers have developed combined or transitional models between the different fouling modes (Bolton et al., 2006; Ho and Zydney, 2006; Iritani, 2013; Tien et al., 2014) and modified the models for more realistic scenarios (Cheng et al., 2011; Tien and Ramarao, 2011; Xiao et al., 2019a). For the temporal change of foulant deposition amount, the process of foulant adsorption onto the membrane surface or pore walls can be described by dynamic adsorption models (in a linear or langmuir mode for example) (Nakamura and Matsumoto, 2006; Mu et al., 2018), and the process of foulant gel/cake layer growth can be described by mass transfer models in the CP boundary layer in relation to hydrodynamic conditions (e.g., crossflow shear rate) (Wang and Li, 2012; Xiao et al., 2013). Mechanism models are used to quantify the membrane-foulant and foulant-foulant interactions in terms of free energy (related to equilibrium constant) or force (as the gradient of energy). Non-covalent interaction can be described by interfacial energy theories such as DLVO and XDLVO theories (van Oss, 2003, 2008; Tang et al., 2011), while covalent complexation can be described by the coordination theory (Chermette, 1998; Wang and Waite, 2009). Recently, computational chemistry tools have been introduced into the simulation of the interactions, such as the molecular docking (Liu et al., 2019) and molecular dynamics (Shaikh et al., 2018) for non-covalent interactions, and the density functional theory (DFT) for covalent interactions (Zhang et al., 2018; Chen et al., 2020). Further study could adopt the combined quantum mechanics/molecular mechanics for a combined treatment of the covalent and non-covalent interactions (Gao and Thompson, 1998) and the coarse-grained molecular dynamics or dissipative particle dynamics for macromolecular interactions on a mesoscopic scale (Müller et al., 2006; Marrink et al., 2007).

## Anti-Fouling Strategies Targeted on the Interactions

### Inhibition of Foulant Migration

The migration of foulants toward the membrane surface could be inhibited via hydrodynamic control (regulating CP or hydrodynamic boundary layer) and electrophoretic back transport.

The reverse diffusion of foulant away from the membrane surface can be enhanced by controlling the hydrodynamics near the membrane surface. In UF processes, the enhancement could be achieved by increasing crossflow intensity, injecting gas to the membrane unit to produce secondary flow and wake flow, equipping movable parts to promote turbulence, and applying spiral flow with high rotation frequency to disturb the mass transfer boundary layer (Cabassud et al., 2001; Adach et al., 2002; Zakrzewska-Trznadel et al., 2009; Kondo et al., 2010). In MBR wastewater treatment processes, the hydraulic enhancement includes optimization of membrane aeration intensity, aerator type and layout, membrane module configuration, membrane cassette layout, and upflow/downflow circulation in the membrane tank (Drews et al., 2008; Yan et al., 2016; Wu et al., 2018). Computational fluid dynamics (CFD) reveal that setting baffles in an airlift MBR tank could constraint the upflow surrounding the membrane unit, elevate the average shear force on the membrane surface, and improve the uniformity of shear distribution to mitigate foulant deposition on the membrane surface (Yan et al., 2015, 2016).

Given a low-voltage electric field, electrophoresis of the negatively charged foulants (e.g., sludge bacteria and extracellular biopolymers) may inhibit their transportation toward the membrane surface and counteract the filtration drag-induced compression of the foulant layer (Chen et al., 2007; Akamatsu et al., 2010; Bani-Melhem and Elektorowicz, 2010; Zhang J. et al., 2015). To facilitate the electric effect, some researchers have combined electrode materials with the membrane to fabricate composite functional membranes, such as carbon-based membranes (Ahmed et al., 2016; Manawi et al., 2016) and stainless-steel mesh composite conductive membranes (Huang et al., 2015).

### Membrane Modification for Tuning the Membrane-Foulant Interaction

To diminish the membrane-foulant hydrophobic interaction, hydrophilic modification has been implemented on the membrane substrate material via blending (Guo et al., 2019; Tao et al., 2019) or copolymerization (Sun et al., 2013; Wang S. et al., 2018), the membrane outer surface via hydrophilic (Higuchi et al., 2002; Li et al., 2015) or superhydrophilic grafting/coating (Liang et al., 2014, 2018; Li et al., 2018; Zhao et al., 2018; Ma Z. B. et al., 2019), and the inner pore walls (Liang et al., 2012). The membrane-foulant electrostatic repulsion could be enhanced or electrostatic attraction reduced by modifying the membrane charge properties via, for instance, *in situ* deposition of electrically conductive polymers such as polyaniline, polypyrrole, and polythiophene on the surface or in the pore structure (Zhan et al., 2004; Qiang et al., 2011; Liu et al., 2012; Sun et al., 2018). Applying an electric capacitive carbon material (e.g., activated carbon) as the supporting layer of the UF membrane could also increase the electrostatic repulsion when negatively charged (Liang et al., 2019). The membrane-foulant covalent complexation could be alleviated by reducing the density of carboxyl groups on the membrane surface (Mo et al., 2012; Han et al., 2016). The spatial effects could be regulated by changing the pore morphology (Xiao et al., 2014a; Fan et al., 2018), surface roughness (Hashino et al., 2011; Feng et al., 2017), and surface topography of the membrane (e.g., prism/pyramid/embossing-patterned membranes Won et al., 2016 and hierarchically textured membranes Zhao et al., 2018). In addition, electrocatalytic membranes have been developed to produce reactive species (such as hydroxyl radicals) on the membrane surface *in situ*, thus breaking the membrane-foulant interactions (Yang Y. et al., 2011; Yang et al., 2012; Zheng et al., 2018).

### Foulant Conditioning for Tuning the Foulant-Foulant Interaction

The metal-mediated complexation between foulant molecules depends on the metal ion concentrations in the solution. There must be a critical concentration of the key metal ion (e.g., Ca^2+^ or Fe^3+^), around which the gel layer formed has the lowest permeability and the fouling is the most severe (Mo et al., 2011; Yang N. N. et al., 2011; Zhang et al., 2014). Therefore, decreasing the metal ion concentration below the critical point via chemical precipitation or ion exchange, or increasing the concentration beyond the critical point via chemical dosing or electrolysis, may alleviate the fouling. Ion exchange resins can remove the hardness ions (Apell and Boyer, 2010), and the magnetic ion exchange resin (MIEX) was found effective in removing organics with complexing groups (Son et al., 2005; Wei et al., 2011; Sun et al., 2013). Using Fe as a sacrificial anodic material, the Fe ions can be electrically released into the solution, and this process is controllable *in situ* (Zhang et al., 2014). When the released concentration exceeds a critical point, the organics tend to agglomerate loosely with the ferric/ferrous hydroxide flocs in the solution rather than form a dense gel layer on the membrane surface (Zhang et al., 2014; Zhang J. et al., 2015).

The properties and concentrations of organic foulants can be regulated by pretreatment using the methods of, e.g., coagulation, adsorption, and oxidation among others (Ha et al., 2004; Oh et al., 2007; Williams and Pirbazari, 2007; You et al., 2007; Treguer et al., 2010; Lee et al., 2014; Sun et al., 2019). The efficiency of the pretreatment for fouling mitigation could be related to the type and dosage of the reagent (coagulant, adsorbent, or oxidant), reaction conditions (time, temperature, and hydraulic mixing), solution environment (pH, ionic strength, etc.), and foulant properties (chemical composition, molecular weight, hydrophobicity, charge, etc.). In addition, the foulant components can be biologically regulated. For example, in an MBR coupled with a sequence batch worm reactor, the proteinous fractions of the soluble and colloidal foulants were significantly eaten by the worms (Yu et al., 2012).

### Membrane Cleaning

**Figure 4:**
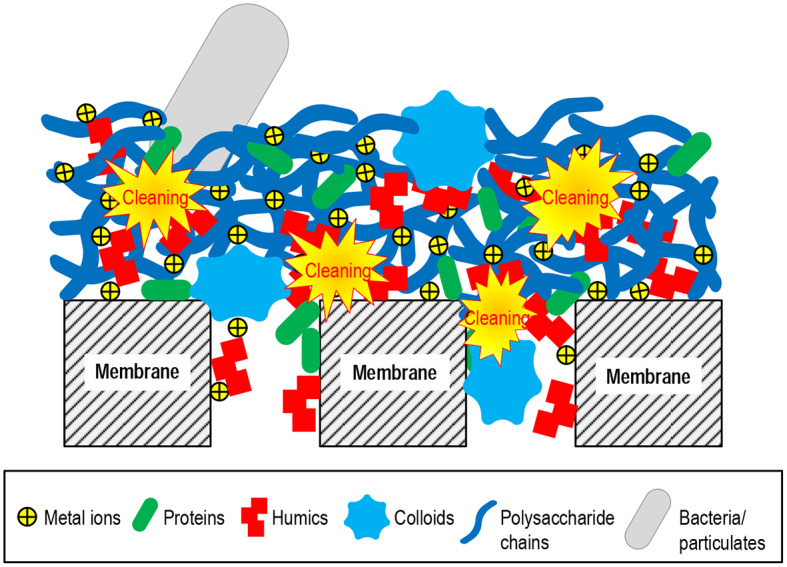
Schematic of the impact of chemical cleaning on the foulant assembly in the membrane pores or on the membrane surface.

Membrane cleaning includes physical cleaning and chemical cleaning. Physical cleaning mainly removes the foulant particles bound by interactions that are relatively weak or sensitive to mechanical stress (e.g., forces related to hydrodynamic conditions). The physical means include hydraulic cleaning (flushing and backwashing), air-assisted cleaning (air lifting, bubbling, and scouring), ultrasonic cleaning, etc. (Lin et al., 2010; Porcelli and Judd, 2010; Shi et al., 2014; Wang et al., 2014). Chemical cleaning dissociates the non-covalent or covalent interactions, or directly breaks the foulant molecular structure, as schematically illustrated in Figure 4. The chemical cleaning agents include acids, alkalis, oxidants, reductants, complexants (chelators), surfactants, and enzymes (Madaeni and Moghadam, 2003; Zondervan and Roffel, 2007; Petrus et al., 2008; Porcelli and Judd, 2010; Shi et al., 2014; Zhang Z. H. et al., 2015). The cleaning mechanisms and reacting targets of the typical agents are summarized in Table 1.

**Table 1:** Cleaning mechanisms and target foulants of the typical membrane cleaning agents.

**Agent type**	**Mechanism**	**Reacting target**	**Typical examples of agents**
Acid	Dissolution (protonation)	Inorganic compounds, and metal ions in organic complexes	HCl; citric acid and oxalic acid (also complexants)
	Acid hydrolysis	Hydrolyzable bonds (e.g., glycoside/peptide/ester bonds) in polysaccharides, proteins, and lipids	HCl
Alkali	Alkaline hydrolysis	Hydrolyzable bonds (e.g., glycoside/peptide/ester bonds) in polysaccharides, proteins, and lipids	NaOH
	Dissolution (ionization)	Organic acids (e.g., humic acids)	NaOH
Oxidant	Oxidative decomposition	A wide range of organics	NaOCl (also alkaline) and H_2_O_2_
Reductant	Reduction	Variable-valence metals [e.g., Fe(III)] in organic complexes and inorganic scales	Ascorbic acid and sodium dithionite
Complexant (chelator)	Complexing extraction	Metal ions in organic complexes and inorganic scales	Na-EDTA and sodium tripolyphosphate (STP)
Surfactant	Dissolution (hydrophobic/philic interfacial activation)	Hydrophobic organics (e.g., proteins, lipids, and humics)	Sodium dodecyl sulfate (SDS) and Tween
Enzyme	Enzyme-catalyzed decomposition	Proteins, lipids, polysaccharides, and other biopolymers	Protease, lipase, carbohydrase, and other hydrolases and oxidases

## Concluding Remarks

This review has outlined the non-covalent, covalent, and spatial aspects of the membrane-foulant and foulant-foulant interactions for membrane organic fouling in MF/UF systems. The dominant interaction(s) may be different in the concentration polarization boundary layer (for the migration of foulant toward the membrane), at the membrane-foulant interface (for the membrane adsorption/pore blocking stage), and in the foulant layer (for the gel/cake layer stage). Notably, there is interplay between different types of the interactions, such as between non-covalent adsorption and steric effects. These interactions correspond closely to the hydrophobic, electrostatic, complexing, and spatial properties of the membrane or foulant. Further fundamental research is required on experimental quantification (e.g., related to the physical/chemical aspects of interfacial phenomena) and theoretical simulation (e.g., mechanistic/statistical models and computational approaches) of the interactions in order to identify the key factors and their influences on the fouling process. While this review is focused on organic fouling, the physical/chemical interactions between organics might be also useful for interpreting the interfacial behavior in biofouling (e.g., adhesion among bacterial cells, extracellular biopolymers, and the membrane). From the standpoint of membrane-foulant and foulant-foulant interactions, targeted anti-fouling strategies could be developed in full accordance with the characteristics of different interactions at different fouling stages.

## Author Contributions

HX and KX wrote the first draft of the manuscript. KX and CW supervised this work. All authors extensively discussed the conception of this work, contributed to manuscript revision, and read and approved the submitted version.

### Conflict of Interest

The authors declare that the research was conducted in the absence of any commercial or financial relationships that could be construed as a potential conflict of interest.
